# Multidrug Resistant *Escherichia coli* among Urinary Samples of Patients with Urinary Tract Infection in the Microbiology Department of a Tertiary Care Center: A Descriptive Cross-Sectional Study

**DOI:** 10.31729/jnma.8160

**Published:** 2023-05-31

**Authors:** Raina Chaudhary, Manoj Pradhan, Sabita Bhatta, Sabnum Shrestha, Nabaraj Adhikari, Yengkokpam Ibotomba Singh

**Affiliations:** 1 Department of Microbiology, Nepalese Army Institute of Health Sciences, Sanobharyang, Kathmandu, Nepal; 2 Kantipur College of Medical Sciences, Sitapaila, Kathmandu, Nepal; 3 Central Department of Microbiology, Tribhuvan University Institute of Science and Technology, Kirtipur, Kathmandu, Nepal.

**Keywords:** *antibiotics*, *Escherichia coli*, *urinary tract infection*

## Abstract

**Introduction:**

Urinary Tract Infection one of the most common and manageable infections still holds its position as a major public health issue worldwide due to an increase in the number of multidrug resistant bacteria. This study aims to find out the prevalence of multidrug resistant *Escherichia coli* among urinary samples of patients with urinary tract infections in the microbiology Department of a tertiary care center.

**Methods:**

A descriptive cross-sectional study was carried out at a tertiary care centre from 8 August 2018 to 9 January 2019. Ethical approval was received from the Institutional Review Committee (Reference number: 123/2018). Clinically suspected cases of urinary tract infection were included in this study. A convenience sampling method was used. Point estimate and 95% Confidence Interval were calculated.

**Results:**

Among 594 patients with urinary tract infections, the prevalence of multidrug resistant *Escherichia coli* was 102 (17.17%) (14.14-20.20, 95% Confidence Interval). Out of which, the production of extended-spectrum beta-lactamase and AmpC beta-lactamase were observed in 74 (72.54%), and 28 (27.45%) isolates respectively. The co-production of extended-spectrum beta-lactamases/AmpC was observed in 17 (16.67%).

**Conclusions:**

The prevalence of multidrug resistant *Escherichia coli* among patients urinary samples of patient with urinary tract infection was lower as compared to the other studies done in similar settings.

## INTRODUCTION

Urinary tract infection (UTI) is a major clinical concern worldwide. *Escherichia coli* is the predominant cause of UTI, accounting for 80% of total infections.^1^ UTI is manageable with antibiotics, however, a rapid increase in resistance towards commonly used antibiotics has been observed in the last decade.^2^ The development of drug resistance results in treatment failure.

The leading mechanism for antibiotic resistance is the production of β-lactamase such as Amp C β-lactamase (ABL) and Extended Spectrum Beta Lactamase (ESBL) among Gram-negative bacteria.^2^ Recently, the production of ESBLs and ABL has limited the use of

β-lactam antibiotics against *E. coli* infections. This has raised a huge concern in the accurate detection of ESBLs and ABL to fight treatment failure.

This study aims to find out the prevalence of multidrug resistant *E. coli* among urinary samples of patients with urinary tract infections in the microbiology Department of a tertiary care center.

## METHODS

A descriptive cross-sectional study was conducted from 8 August 2018 to 9 January 2019 after taking ethical approval from the Institutional Review Committee of the Nepalese Army Institute of Health Sciences, Sanobharyang, Kathmandu, Nepal (Reference number: 123/2018). Clinically suspected cases of urinary tract infection were included in this study. Non-labelled samples, samples over 2 hours of collection without appropriate storage and Foley's catheter tip sample were excluded from the study. Convenience sampling was used. The sample size was calculated using the following formula:


$$\begin{array}{ll} \text{n} & ={\text{Z}}^{2}\times \frac{\text{p}\times \text{q}}{{\text{e}}^{\text{2}}}\\ & =1.96^{2}\times \frac{0.41\times 0.59}{0.04^{2}}\\ & =582 \end{array}$$

Where,

n = minimum required sample size
Z = 1.96 at 95% Confidence Interval (CI)
p = prevalence of MDR producers taken from previous study, 41.1%^3^
q = 1-p
e = margin of error, 5%

The calculated sample size was 582. However, 594 patients were included in the study. In accordance with the established protocol, clean-catch midstream urine was collected in a sterile container. The tube was clamped for a number of minutes before the sample is extracted from it for individuals who have an indwelling urine catheter. The samples were immediately submitted to the lab, where they are inoculated using a flame-sterilized nichrome wire loop (internal diameter of 4 mm containing 0.01 ml) into cystine-lactose-electrolyte-deficient (CLED) agar plates.

A semi-quantitative standard loop method was used for urine cultures. The plates were incubated at 37°C and were observed for bacterial growth after 24 hour. The bacteria were identified according to colony characteristics, Gram's staining and biochemical properties. Bacterial colonies of more than 10^5^ colony-forming units (CFU) per ml of urine were considered to represent significant bacteriuria. These are then subjected to antibiogram testing by Kirby-Bauer's disc diffusion method using Mueller-Hinton agar for identifying bacterial susceptibility and resistance according to Clinical Laboratory Standard Institute (CLSI) guidelines.^4^

Multidrug resistant (MDR) *E.coli* isolates were selected. For the detection of MDR, at least nine classes of antibiotics were used and results were defined as MDR when an isolate was non-susceptible to ≥1 agent in >3 antimicrobial categories (2012).^5^

The MDR isolates were screened for possible ESBL production using ceftriaxone (30 μg), Ceftazidime (30 μg) and cefotaxime (30 μg) (CLSI 2016). According to the guidelines, isolates showing Ceftazidime < 22 mm, cefotaxime < 27 mm, and Ceftriaxone < 25 mm are the possible ESBL-producing strains.

The screen-positive isolates i.e. suspected ESBL producers were subjected to a phenotypic confirmatory test (Combined Disk Assay) in Mueller-Hinton agar using guidelines and interpretive criteria of CLSI ceftazidime (30 μg) with and without clavulanic acid (10 μg) discs were used for ESBL detection. An increase in zone diameter of ≥5 mm around the Ceftazidime disc with clavulanic acid compared to ceftazidime alone was concluded as confirmed ESBL producers.^4^

The screening test for the suspected isolate of AmpC β-lactamase producer was done using Cefoxitine 30 μg whereas a phenotypic confirmation test was carried out on Muller-Hinton agar using Cefoxitine (30 μg) with or without cloxacillin (200 μg). An increase in zone diameter of ≥ 4 mm around the Cefoxitin disc with cloxacillin compared to cefoxitin alone was concluded as confirmed AmpC producers.^6^

Quality control of laboratory equipment, reagents and media was carried out regularly. Mueller-Hinton agar and the antibiotic discs were checked for their lot number, manufacture and expiry date, and proper storage. For the standardization of the Kirby-Bauer test and for performance testing of antibiotics and MHA, control strains of *E. coli* (ATCC 25922) and S. *aureus* (ATCC 25923) were tested primarily. Quality of sensitivity tests was maintained by maintaining the thickness of Mueller-Hinton agar at 4 mm and the pH at 7.2-7.4.

Data was entered in Microsoft Excel 2016 and analysis was done using IBM Statistics SPSS 18.0. Point estimate and 95% CI were calculated.

## RESULTS

Among 594 patients with urinary tract infections, the prevalence of multidrug resistant Escherichia coli was 102 (17.17%) (14.14-20.20, 95% CI). These 102 isolates were further tested for P Lactamase production. The antibiotic susceptibility pattern of MDR *E. coli* is shown in (Table 1).

**Table 1:** Antibiotic susceptibility pattern of MDR *E. coli* isolates (n= 102).

Antibiotics used	Resistant n (%)
**First line of antibiotics**	
Amoxycillin	102 (100)
Cefixime	102 (100)
Cefotaxime	102 (100)
Ceftriaxone	102 (100)
Ciprofloxacin	99 (97.05)
Cotrimoxazole	88 (86.27)
Levofloxacin	84 (82.35)
Nitrofurantoin	13 (12.74)
**Second line of antibiotics**	
Ceftazidime	102 (100)
Meropenem	96 (94.11)
Cefoperazone/Sulbactam	60 (58.82)
Doxycycline	59 (57.84)
Piperacillin/Tazobactam	36 (35.29)
Gentamycin	21 (20.58)
Imipenem	12 (11.76)
Amikacin	11(10.78)

Among 102, 57 (55.88%) were found to be ESBL producers only, 11 (10.78%) produced Amp C only, 17 (16.67%) were co-producers and 17 (16.67%) were nonproducers. Also among the 102 MDR *E. coli* isolates, 77 (75.49%) isolates were resistant to Cefoxitin (AmpC screening positive), in which 28 (27.45%) of isolates were found to be Amp C β-lactamase producers (Table 2).

**Table 2:** Antibiotic resistance profile among β-lactamase producers (n= 102).

Antibiotic used	ESBL n (%)	AmpC n (%)	ESBL+ AmpC n (%)
Amoxycillin	57 (55.88)	11 (10.78)	17 (16.67)
Cefotaxime	57 (55.88)	11 (10.78)	17 (16.67)
Ceftriaxone	57 (55.88)	11 (10.78)	17 (16.67)
Ceftazidime	57 (55.88)	11 (10.78)	17 (16.67)
Cefixime	57 (55.88)	11 (10.78)	17 (16.67)
Ciprofloxacin	55 (53.92)	11 (10.78)	17 (16.67)
Cotrimoxazole	48 (47.06)	10 (9.80)	14 (13.73)
Nitrofurantoin	5 (4.90)	2 (1.96)	2 (1.96)
Levofloxacin	46 (45.10)	11 (10.78)	12 (11.76)
Amikacin	1 (45.10)	6 (5.88)	1 (0.98)
Gentamycin	8 (7.84)	6 (5.88)	1 (0.98)
Cefoperazone + Sulbactum	27 (26.47)	11 (10.78)	8 (7.84)
Piperacillin + Tazobactum	8 (7.84)	4 (3.92)	10 (9.80)
Meropenem	53 (51.96)	11 (10.78)	15 (14.71)
Imipenem	-	8 (7.84)	1 (0.98)
Doxycycline	32 (31.37)	9 (8.82)	8 (53.92)

## DISCUSSION

Of 594 total isolates of *E. coli,* 102 (17.17%) isolates were multidrug resistant. The result seems much less than the previous study by 29.62%.^7^ This discrepancy may be due to the difference in the time frame and inclusion of a large number of samples in the later study. Usually, the high prevalence of MDR could be explained by the fact that drugs are easily available without a doctor's prescription from a pharmacy and in developing countries like Nepal where self-medication is a common practice and this might probably be a major cause of antibiotic resistance in clinical isolates. Expired antibiotics, self-medication, and inadequate hospital control measures can as well promote the development of resistance in clinical isolates. Significantly high rates of resistance to these commonly used oral antibiotics make these agents clinically useless for the empirical treatment of infections caused by *E. coli,* such as urinary tract infections (UTI). Resistance to ampicillin, cephalosporins, quinolones, aminoglycosides, cotrimoxazole, chloramphenicol and even partial resistance to nitrofurantoin has been detected in hospital influents.^8^

In our study MDR *E. coli* isolates were analyzed for resistance ability against a second line of antibiotics and were found to resist most of the antimicrobial agents. Among the antibiotics, imipenem was the most effective from the urine sample followed by amikacin and nitrofurantoin indicating these as the most potent. This finding was similar to other studies.^9,10^ The explanation for amikacin and imipenem is probably the fact that these are very powerful drugs used only in a hospital setting and not as first-line therapy and therefore, have lower selective pressure due to their restricted use. It is interesting to note that the antibiotics imipenem and amikacin are only available for intravenous administration and provided on prescription only. Hence, the route of administration of these antibiotics may have reduced their misuse which led to the reduction in the emergence of resistant bacterial strains.

The high level of drug resistance seen among *E. coli* is mediated by β-lactamases. In addition to this mechanism, there are more than seven efflux systems in *E. coli* that can export structurally unrelated antibiotics; these multidrug-resistance efflux pump (MDR pump) systems contribute to intrinsic resistance for toxic compounds such as antibiotics, antiseptics, detergents, and dyes.^11^

Out of 102 MDR *E. coli* isolates suspected the possible β-lactamase producer, 85 isolates were found to be producing different classes of β-Lactamases while 17 isolates did not produce any β-Lactamases as. Similarly, 77 MDR *E. coli* (75.49%) isolates were resistant to cefoxitin (AmpC screening positive). Of which 28 (27.45%) were found to be Amp C β-lactamase producers in which 11 (10.78%) isolates were found to be AmpC producers only. In a previous study, the prevalence of AmpC production among *E. coli* isolates was found to be 19.8%, 24.0% and 8.3% respectively.^12,13^ In this study, 53.7% of Cefoxitin-resistant isolates were not positive for AmpC production by combined disc diffusion test. Two reasons could explain this observation. Firstly, the inability of current phenotypic tests to accurately detect transferable AmpC β-lactamase does not allow for the differentiation of multiple AmpC enzymes. Second, it is possible that there is a limit to the amount of AmpC β-lactamase that a bacterial cell can accommodate and still be a viable pathogen. Cefoxitin resistance in this type of AmpC-negative isolates could be due to decreased outer membrane permeability (porin mutation).^14-15^

Both ESBL and AmpC were detected in 17 (16.70%) isolates. The previous study reported only 8.8% and 8.0% of isolates co-producing ESBL and AmpC which is comparatively lower than our findings.^12,13^

In the present study, ESBL producers showed complete resistance to amoxicillin; third-generation cephalosporins (cefixime, cefotaxime, ceftriaxone, ceftazidime), whereas high resistance was observed against fluoroquinolones, meropenem, cotrimoxazole, doxycycline, while the isolates were susceptible to imipenem, amikacin and nitrofurantoin. Isolates were found to be sensitive to piperacillin/tazobactam (91%) and cefoperazone/sulbactam (78%). In addition, multiple β-lactamases producers were also found to be resisting most of the antibiotics. Simultaneous production of various β-lactamases in these isolates reflects the increased ability of these isolates against antibacterial agents; therefore, this can cause serious problems in future in the treatment of infections especially nosocomial infections of such isolates. ESBL/AmpC co-producers were sensitive to nitrofurantoin, aminoglycosides and imipenem leaving remaining antibiotics resistant.

Only phenotypic studies were performed for the detection of ESBL and AmpC detection. Molecular detection of ESBL and AmpC encoding genes was not performed.

## CONCLUSIONS

Our study showed that the prevalence of multidrug resistant *E. coli* among urinary samples of patients with urinary tract infections was lower as compared to the other studies done in similar settings. Although the overall prevalence of MDR *E. coli* was lower, the number of ESBL producers and AmpC producers is on the higher side. In our study, we found that AmpC and ESBL a major causes of the MDR mechanism of *E. coli.*

Support for greater antibiotic stewardship and enhanced UTI treatment recommendations are necessary to change this. Thus, controlling antibiotic-resistant bacteria and subsequent infections more efficiently necessitates the prudent and responsible use of antibiotics.
